# Co-financing for viral load monitoring during the course of antiretroviral therapy among patients with HIV/AIDS in Vietnam: A contingent valuation survey

**DOI:** 10.1371/journal.pone.0172050

**Published:** 2017-02-15

**Authors:** Quyen Le Thi Nguyen, Long Hoang Nguyen, Bach Xuan Tran, Huong Thi Thu Phan, Huong Thi Le, Hinh Duc Nguyen, Tho Dinh Tran, Cuong Duy Do, Cuong Manh Nguyen, Vu Thi Minh Thuc, Carl Latkin, Melvyn W. B. Zhang, Roger C. M. Ho

**Affiliations:** 1 Institute for Global Health Innovations, Duy Tan University, Da Nang, Vietnam; 2 School of Medicine and Pharmacy, Vietnam National University, Hanoi, Vietnam; 3 Institute for Preventive Medicine and Public Health, Hanoi Medical University, Hanoi, Vietnam; 4 Department of Immunology and Allergy, National Otolaryngology Hospital, Hanoi, Vietnam; 5 Authority of HIV/AIDS Control, Ministry of Health, Hanoi, Vietnam; 6 Department of Hepatobiliary Surgery, Viet-Duc Hospital, Hanoi, Vietnam; 7 Department of Infectious Diseases, Bach Mai Hospital, Hanoi, Vietnam; 8 Department of International Affairs, Ministry of Health, Hanoi, Vietnam; 9 Bloomberg School of Public Health, Johns Hopkins University, Baltimore, MD, United States of America; 10 Biomedical Global Institute of Healthcare Research & Technology (BIGHEART), National University of Singapore, Singapore, Singapore; 11 Department of Psychological Medicine, Yong Loo Lin School of Medicine, National University of Singapore, Singapore, Singapore; Katholieke Universiteit Leuven Rega Institute for Medical Research, BELGIUM

## Abstract

**Background:**

Viral load testing is considered the gold standard for monitoring HIV treatment; however, given its high cost, some patients cannot afford viral load testing if this testing is not subsidized. Since foreign aid for HIV/AIDS in Vietnam is rapidly decreasing, we sought to assess willingness to pay (WTP) for viral load and CD4 cell count tests among HIV-positive patients, and identified factors that might inform future co-payment schemes.

**Methods:**

A multi-site cross-sectional survey was conducted with 1133 HIV-positive patients on antiretroviral therapy (ART) in Hanoi and Nam Dinh. Patients’ health insurance coverage, quality of life, and history of illicit drug use were assessed. A contingent valuation approach was employed to measure patients’ WTP for CD4 cell count and viral load testing.

**Results:**

HIV-positive patients receiving ART at provincial sites reported more difficulty obtaining health insurance (HI) and had the overall the poorest quality of life. Most patients (90.9%) were willing to pay for CD4 cell count testing; here, the mean WTP was valued at US$8.2 (95%CI = 7.6–8.8 US$) per test. Most patients (87.3%) were also willing to pay for viral load testing; here, mean WTP was valued at US$18.6 (95%CI = 16.3–20.9 US$) per test. High income, high education level, and hospitalization were positively associated with WTP, while co-morbidity with psychiatric symptoms and trouble paying for health insurance were both negatively related to WTP.

**Conclusions:**

These findings raise concerns that HIV-positive patients in Vietnam might have low WTP for CD4 cell count and viral load testing. This means that without foreign financial subsidies, many of these patients would likely go without these important tests. Treating psychiatric co-morbidities, promoting healthcare services utilization, and removing barriers to accessing health insurance may increase WTP for monitoring of HIV/AIDS treatment among HIV+-positive Vietnamese patients.

## Introduction

Regular monitoring of HIV-positive patients on antiretroviral therapy (ART) is a critical component of HIV/AIDS care. Through monitoring, non-adherence or deterioration of immune function during the course of the illness can both be detected. Monitoring can promptly inform clinicians if patients have poor responses to treatment, reducing the risk of development of resistance to ARTs, transmission of HIV to others, and premature morbidity and mortality due to the virus [1]. Furthermore, promotion of regular viral load and CD4 cell count monitoring is an important extension of existing ART programs, especially in developing countries [2].

For the past two decades, monitoring patients’ CD4 cell count has been considered the primary target of routine monitoring during the course of ART [2]. The CD4 cell count is used to evaluate HIV disease progression and ART response, as well as predict the likelihood of mortality [3–7]. In addition, a reduction of CD4 cell count to below 200 cells/mm^3^ indicates increasing risk for opportunistic infections [7,8]. In terms of HIV treatment, previously, patients were started on ART when their CD4 counts went below 500 cells/mm^3^ [9]. However, current guidelines suggest that ART should be started as soon as a patient receives a diagnosis of HIV, regardless of his or her CD4 cell count [10].

Since 2010, the World Health Organization (WHO) has recommended including HIV viral load (plasma HIV RNA) testing as part of treatment monitoring [11]. The viral load test detects the existence of HIV RNA thereby determining the number of copies of the virus in a patient's blood. Empirical evidence shows that the CD4 cell count is not as accurate or sensitive as the viral load test in monitoring disease progression [12–14]. Viral load testing identifies treatment failure earlier and prevents the unimportant second or third-line regimes switching. For example, one multi-national study revealed that among those patients whose treatment failure was detected by CD4 cell count and viral load test, 70% of those patients might have switched to second-line treatment regimens unnecessarily without confirmation of viral load [15]. Therefore, the WHO asserts that where possible, HIV viral load should be used in lieu of CD4 cell counts to monitor treatment response [10].

Each nation across the globe has differing availability of viral load testing. Viral load testing is considered the gold standard test for monitoring HIV-positive patients, and such testing is very common in wealthy countries [16]. However, due to the high medical costs involved in viral load testing, its application is limited in developing countries, except those countries receiving financial support from outside sources [17]. Therefore, in the majority of resources-limited nations, viral load and CD4 cell count testing continue to exist together as valid measurements for monitoring HIV treatment [18].

Vietnam has an estimated 100,000 HIV-positive patients currently receiving ART [19]. In 2009, the Vietnamese Ministry of Health published a standard national guideline for HIV treatment and care, requiring that HIV-positive patients have to measure their CD4 cell count and HIV viral load every six months [20]. Currently, Vietnam is facing a financial crisis with regards to maintaining its HIV/AIDS monitoring program, due to withdrawal of foreign subsidies for HIV/AIDS initiatives over the past several years. Currently, subsidies from the Vietnamese government only covered approximately 6–12% of the total operational costs for the national HIV/AIDS program [21]. HIV-positive patients in Vietnam may not be able to receive recommended monitoring tests on a regular basis, because of lack of financial subsidies from the Vietnamese government and the decrease in foreign donations.

The Vietnamese government has considered alternative financial mechanisms to help HIV-positive patients afford regular CD4 cell count and viral load testing. These alternatives include universal health insurance (HI) and/or co-payments for HIV-positive patients. There is a dearth of research regarding the concept of willingness to pay (WTP) for CD4 cell count and viral load tests among HIV-positive patients in Vietnam. This study aimed to understand HIV-positive patients’ WTP for CD4 cell counts and viral load tests as part of their HIV/AIDS disease monitoring program in Vietnam. We also sought to identify factors associated with patients’ increasing or decreasing WTP for these monitoring services.

## Materials and method

### Study setting and participants

A cross-sectional survey was conducted from January to August 2013 in Hanoi and Nam Dinh, two epicenters providing HIV/AIDS monitoring and treatment services in northern Vietnam. This study was approved by the Institutional Review Board of the Vietnam Administration of HIV/AIDS control. We purposively selected eight outpatient clinic sites in Hanoi and Nam Dinh as our study sites based on the following inclusion criteria: 1) the clinics had to be representative of typical public health systems in Vietnam (including central-; provincial- and district- levels); 2) the clinics had to have the capacity to provide ART; and 3) the clinics had to implement their ART programs using the official guidelines from the Vietnamese Ministry of Health [22]. Eight outpatient clinics—five from Hanoi and three from Nam Dinh—were selected, including one national hospital (Bach Mai Hospital), one provincial hospital (Nam Dinh provincial hospital), one provincial center (Nam Dinh Provincial AIDS Control Centre) and five district health centers (Hoang Mai, Long Bien, Dong Anh, Ha Dong, Xuan Truong). Table 1 displays the characteristics of those centers that met the inclusion criteria and were included in the study.

**Table 1:** Characteristics of the centers providing HIV/AIDS monitoring and treatment that were included in this study.

Name	Level	Location	Other services
Bach Mai Hospital	Central	Hanoi	ART, General Health
Nam Dinh provincial hospital	Provincial	Nam Dinh	ART, General Health
Nam Dinh Provincial AIDS Center	Provincial	Nam Dinh	MMT, ART
Ha Dong Hospital	Provincial	Hanoi	ART, General Health
Xuan Truong district health center	District	Nam Dinh	ART, MMT, General Health
Dong Anh district health center	District	Hanoi	ART, General Health
Hoang Mai district health center	District	Hanoi	ART, General Health
Long Bien district health center	District	Hanoi	ART, MMT, VCT, General Health

**ART:** Antiretroviral treatment; **MMT:** Methadone maintenance treatment; **VCT:** Voluntary counseling and testing

The inclusion criteria for research participants included 1) being at least 18 years of age; 2) having a confirmatory test indicating that the patient is HIV-positive; 3) receiving ART from one of the clinics listed above; 4) having no major cognitive impairment; and 5) agreeing to participate and providing written informed consent. People suffering from serious illness during the recruitment process were excluded.

Eligible patients were invited into a small counseling room. They were introduced to the purpose of study, the benefits and drawbacks of participating, and were then asked to join the study. If they agreed, patients signed a written informed consent. We ensured patients of the confidentiality of their participation in the study. The consent process took place in a comfortable room with restricted access, allowing patients privacy as they decided whether or not to join the project.

Convenience sampling was used to recruit at least 200 HIV+ patients at the national center and 100 patients at provincial and district health centers levels. Participation rates ranged from 80–90% in all clinics, among those patients who met the inclusion criteria and were asked to join. Reasons for deciding not to participate included discomfort, having busy work or insufficient physical health. A total of 1133 patients were enrolled in the study.

### Measures and instruments

Patients completed a 20-minute face-to-face interview. Patients first completed a structured questionnaire which asked them for the following information:

#### Socioeconomic characteristics

Items surveyed here included age, gender, education level, marital status, employment status and monthly household income per capita. Among all patients surveyed, income was then divided into five quintiles from “poorest” to “richest”.

#### Household expenditures

Household monthly spending was measured in two parts: recurring/regular expenditure (namely food, clothes, rent, utility, education, etc.) over the last 30 days, and non-recurring/irregular expenditure (e.g. health care, furniture, spending for special occasions, travel, construction, etc.) over the last 12 months. Catastrophic health expenditure was also measured. “Catastrophic” in this instance means paying for a health service that exceeds 10% of total household expenditures [23,24].

#### Health status

We applied the EuroQol—5 Dimensions– 5 levels (EQ-5D-5L) instrument to measure patients’ quality of life in five domains: mobility, self-care, usual activities, pain/discomfort, and anxiety/depression. Each domain was assessed using a Likert scale, with five levels of responses ranging from “no problems” to “extreme problems” [25].

#### History of drug abuse and rehabilitation

We collected information about patients’ history of intravenous drug abuse (IVDA), current illicit drug use, the number of episodes of drug rehabilitation, age of onset of illicit drug abuse, years of illicit drug abuse, and duration of Methadone Maintenance Treatment (MMT) (if any).

#### Accessing health insurance

Patients were asked whether they had health insurance, the types of health insurance they had acquired, the number of people in their family that had health insurance, and any barriers to accessing health insurance (such as lack of information, difficulty to access/use/pay for health insurance, and mismatches in their expectations versus actual health insurance policy).

### Preference and willingness to pay for monitoring HIV/AIDS treatment

Before participants were asked to determine their WTP for CD4 cell count and viral load monitoring, they were provided with the following information: 1) the key effects of the monitoring tests; 2) recommended frequency of taking the tests (every six months; and 3) a brief explanation of the impact of reduction in foreign financial subsidy for those tests. To ensure that there was no bias involved when measuring patients’ WTP, patients were told to assume that the cost of tests was not going to be covered by any financial support, including health insurance, in the future.

A contingent valuation (CV) approach using double-bounded dichotomous choice (DBDC) questions backed by an open-ended (OE) question was utilized to elicit patients’ WTP. This method seeks to reflect the behavior of consumers in a regular market [26], using consecutive questions to elicit patients' true WTP. The literature suggests this technique provides more efficient estimations of WTP than those using only a single question [27]. The information gleaned from the CV approach enables policy-makers to plan financing schemes that can fill the gap between the amount patients can pay for a particular service, and the actual cost of the service [28].

To determine the starting bids, we investigated the actual price of CD4 cell count and HIV viral load testing in several hospitals that delivered testing services, including Bach Mai Hospital and the National Hospital of Tropical Diseases. In 2013, overall unavailability of the proper resources for testing, such as insufficient testing machine or manpower, caused the prices of the tests to be quite high: the prices in the real market were 1.9 million VND (~ US$ 95) per viral load test and 300 thousand VND (~US$ 15) per CD4 cell count test.

To measure participants’ WTP for viral load testing, they were asked a maximum of four questions. Each question had three options: “Yes/No/I don’t know”. Firstly, respondents were asked to answer the following question: “Would you be willing to pay for biannual viral load monitoring tests?”. If patients said “yes”, they were then asked: “Would you be willing to pay for biannual viral load tests if the price were US$ 100 (~2 million VND) per test?” If the respondents answered “yes” to the first bid offered, the interviewer then doubled the bid. If the answer was “no”, the bidding price was reduced by one half. Finally, patients were asked an OE question: “What is the maximum price you would be willing to pay for the biannual monitoring tests, including viral loads?”.

If a patient answered “I don’t know” to any question, he or she would be immediately dropped from the sample in the data analysis process. This strategy was employed in order to decrease protest bids, which could introduce bias [29–31]. This bias could occur when a person feels that placing monetary value on a public good is unreasonable, or when he or she believes that this good should be provided freely [29].

**Fig 1:**
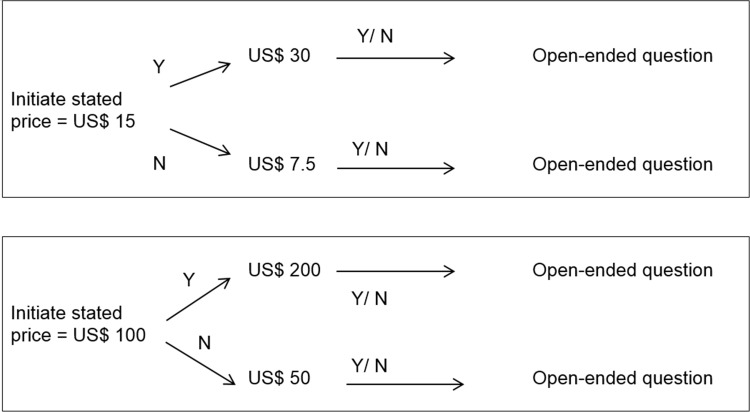
The Bidding process, based on the contingent valuation model. (A) Bidding process to measure WTP for CD4 cell count; (B) Bidding process to measure WTP for Viral load test. *Note*: *N indicates unwillingness to pay; Y indicates willingness to pay.

Assessment of WTP for CD4 cell count testing underwent a similar multi-question process and started at US$ 15 (~ 300 thousand VND, 2013 exchange rate). The processes for measuring patients’ WTP for HIV viral load and CD4 cell count testing is summarized in **Fig 1A** and **Fig 1B**.

### Data analysis

STATA version 12 (Stata Corp. LP, College Station, USA) was used to analyze the data. We used the analysis of variance (ANOVA), Kruskal-Wallis and Chi-square tests to measure differences in characteristics among patients at the three health care levels (central, provincial, and district). A value of p<0.05 was considered statistically significant.

The CV method assumes that the variable *Y*_*i*_ represents the WTP for testing of person *i* with characteristics *X*_*i*_, following the model [32]:

$$Y_{i}=X_{i}\beta +\varepsilon_{i}$$

where *ε*_*i*_ has a normal distribution with a mean of zero. Since we observe respondents’ WTP indirectly via their responses to the CV questions, we know the value of *Y*_*i*_ to lies within the interval [*Y*_*i1*_*; Y*_*i2*_]. The individual likelihood contribution is displayed as:

$$Pr(Y_{i1}\leq Y_{i}\leq Y_{i2}=Pr(Y_{i1}<X_{i}\beta <Y_{i2})$$

When the data is right-censored (meaning patients were not willing to pay the initial price, resulting in missing data regarding WTP data for the double bid and thus an unknown upper bound), the likelihood contribution is Pr(*X*_*i*_*β* + *ε*_*i*_ ≤ *Y*_*i*2_)

If the data is left-censored (for people were willing to pay the initial price, leading to missing data regarding WTP for the half bid and thus an unknown lower bound), the likelihood contribution is Pr(*Y*_*i*1_ ≤ *X*_*i*_*β* + *ε*_*i*_).

In this study, we applied both the DBDC method and OE follow-up questions. Traditionally, each respondent’s WTP was classified as left- or right- censored data, based on their answer for each WTP bid. WTP measured by the DBDC-OE method, however, allows for the combination of censored and uncensored data. For example, suppose a patient was willing to pay US$ 100 for a viral load test, subsequently answered “Yes” for the next bid of US$ 200 for the same test, and gave a final WTP maximum value of US$250 for the OE question. In DBDC, the lower bound would be US$100 and the upper bound would be US$200, while in DBDC-OE, the lower bound is US$100 and the upper bound is US$250. The DBDC-OE approach enhances the accuracy of WTP estimates, relative to the DBDC approach alone. Previous literature has suggested that DBDC-OE has a lower magnitude of starting point bias and incentive incompatibility than DBDC alone [32,33].

Interval regression was employed to estimate the maximum likelihood function. Interval regression can estimate the probability of the latent variable lying within a specific interval. The dependent variables in the interval model thus included both upper-bound and lower-bound variables. The results were used to calculate the mean amount that the patients were willing to pay for the CD4 cell count and viral load testing.

These interval models were also used to identify factors associated with patients’ WTP, by examining previously defined socioeconomic characteristics (gender, age, education, employment status, marital status, household income per capita, catastrophic health expenditure); health status (HRQOL regarding EQ5D5L); illicit drug use behavior (history of injection drug abuse, current injection drug use, history of drug rehabilitation, age of onset of illicit drug abuse, years of illicit drug use, and duration of MMT); access to health insurance (having health insurance or not, the types of available health insurance, the number of people in the participant’s family with health insurance, availability of information regarding health insurance, ease of access/use/pay with regards to health insurance, and level of agreement between patient expectations and health insurance policies). Reduced models were built based on stepwise backward strategies, with p<0.2 being the threshold used for removing non-significant variables.

### Ethical approval

The protocol described above was approved by the Vietnam Administration of HIV/AIDS Control. Data collection procedures were approved by the leaders of each clinic included in the study. Patient information was kept protected and confidential. Patients were informed that they could withdraw from the interview at any time without any negative impact on the health care they would receive at the clinic.

## Results

### Background characteristics of study participants

**Table 2** summarizes the socio-economic demographic data of the HIV-positive patients included in this study. Among the 1133 HIV-positive patients that participated, the majority were men (58.7%), lived with a spouse/partner (61.2%), and did not belong to any religion (88.4%). There were significant differences in gender distribution and marital status among recruitment sites. Participants recruited from central HIV/AIDS clinics were mroe likely to be male (65%) and were also more likely to live with a spouse/partner (64.6% in the central clinics vs 55% in the provincial clinics). More patients were between the ages of 30 and 35 years (336.5% of participants) than any other age group. Mean monthly household income per capita was 2.4 million VND (SD = 2.8 million VND).

**Table 2:** Socio-demographic characteristics of respondents based on recruitment site.

	HIV/AIDS services								P value
	Central		Provincial		District		Total		
	N	%	N	%	N	%	N	%	
**Age groups**									
18- <25	7	2.5	5	1.8	11	1.9	23	2.0	0.77
25-<30	38	13.7	34	12.5	77	13.2	149	13.2	
30- <35	102	36.8	109	39.9	202	34.7	413	36.5	
35- <40	65	23.5	69	25.3	174	29.9	308	27.2	
40- <45	34	12.3	31	11.4	67	11.5	132	11.7	
> = 45	31	11.2	25	9.2	52	8.9	108	9.5	
**Gender**									
Male	180	65.0	151	55.3	334	57.3	665	58.7	0.04
Female	97	35.0	122	44.7	249	42.7	468	41.3	
**Education**									
<Elementary	41	14.8	78	28.6	113	19.4	232	20.5	<0.01
Secondary	82	29.6	115	42.1	221	37.9	418	36.9	
High school	99	35.7	70	25.6	193	33.1	362	32.0	
>Vocational	55	19.9	10	3.7	56	9.6	121	10.7	
**Marital status**									
Single	46	16.6	43	15.8	80	13.7	169	14.9	0.04
Living with spouse/partner	179	64.6	150	55.0	364	62.4	693	61.2	
Divorced/widow	52	18.8	80	29.3	139	23.8	271	23.9	
**Religion**									
No	242	87.4	245	89.7	514	88.2	1001	88.4	0.67
Yes	35	12.6	28	10.3	69	11.8	132	11.7	
**Employment**									
Unemployed	51	18.4	60	22.0	121	20.8	232	20.5	<0.01
Self-employed	109	39.4	95	34.8	265	45.5	469	41.4	
White collar worker	35	12.6	10	3.7	35	6.0	80	7.1	
Blue-collar worker	42	15.2	99	36.3	141	24.2	282	24.9	
Other jobs	40	14.4	9	3.3	21	3.6	70	6.2	
**Catastrophic health expenditure within the past 12 months**									
No	205	74.0	204	74.7	459	78.7	868	76.6	0.22
Yes	72	26.0	69	25.3	124	21.3	265	23.4	
	**Mean**	**SD**	**Mean**	**SD**	**Mean**	**SD**	**Mean**	**SD**	
Monthly household income per capita (in millions of VND)	3.0	3.8	1.4	1.4	2.5	2.7	2.4	2.8	<0.01

More patients had completed secondary education (36.9%) than any other education level. There were significant differences in education levels between different recruitment sites. Participants recruited from central HIV/AIDS clinics had the highest level of education, with 35.7% having completed higher levels of education (compared with 25.6% of patients from the provincial clinics). Most patients were self-employed (41.4% of participants), followed by those who were workers/farmers (24.9% of participants). There were significant differences in patients’ employment status between different recruitment sites. Participants recruited from district HIV/AIDS clinics were more likely to be self-employed (45.5% vs 34.8% in provincial HIV/AIDS clinics). There were 23.4% of patients that had faced catastrophic health expenditures within the last 12 months.

### Physical and mental health status of participants

**Table 3** shows the physical and mental health status of the HIV-positive patients included in this study. The results indicate that about 20.5% of participants had problems with mobility, 9.7% had problems with self-care, and 16.6% had trouble engaging in usual daily activities. More than one-third of respondents endorsed pain/discomfort, and approximately one half patients had anxiety/depression. HIV+-positive patients receiving care from provincial HIV/AIDS clinics demonstrated significantly more problems with mobility, self-care, usual activities, pain/discomfort and anxiety/depression (p<0.01) than participants from district or central HIV/AIDS clinics. Approximately one-third (32.1%) of the patients had a history of IVDA and 6.2% were current illicit drug users. Among them, mean age at first drug use was 22.8 (SD = 5.8) years and the average duration of illicit drug use was 14.2 (SD = 5.0) years. Few participants (2.7%) had received more than five episodes of drug rehabilitation. HIV-positive patients receiving treatment from provincial HIV/AIDS clinics participated in a significantly higher number of episodes of drug rehabilitation and had been on MMT for a longer duration (p<0.01); these patients were also the most likely to report having no family member with health insurance (20.9% of patients from provincial HIV/AIDS clinics, versus 14.2% of patients from central HIV/AIDS clinics, p<0.01).

**Table 3:** Participants’ physical and mental health status, intravenous drug abuse history, and drug abuse treatment history.

	HIV/AIDS services								P value
	Central		Provincial		District		Total		
	N	%	N	%	N	%	N	%	
**EQ5D5L**									
Having problems with mobility	30	10.8	81	29.7	121	20.8	232	20.5	<0.01
Having problems with self-care	21	7.6	47	17.2	42	7.2	110	9.7	<0.01
Having problem conducting usual activities	30	10.8	67	24.5	91	15.6	188	16.6	<0.01
Pain/Discomfort	69	24.9	146	53.5	212	36.4	427	37.7	<0.01
Anxiety/Depression	82	29.6	177	64.8	250	42.9	509	44.9	<0.01
**History of IVDA**									
No	201	72.6	188	68.9	380	65.2	769	67.9	0.09
Yes	76	27.4	85	31.1	203	34.8	364	32.1	
**Current status of IVDA**									
No	82	92.1	86	91.5	209	95.4	377	93.8	0.32
Yes	7	7.9	8	8.5	10	4.6	25	6.2	
**No. of episodes of drug rehabilitation**									
0	212	76.5	184	67.4	383	65.7	779	68.8	0.02
1–5	58	20.9	84	30.8	181	31.1	323	28.5	
>5	7	2.5	5	1.8	19	3.3	31	2.7	
	**Mean**	**SD**	**Mean**	**SD**	**Mean**	**SD**	**Mean**	**SD**	
Age at onset of illicit drug use	21.9	5.3	23.6	5.8	22.8	5.9	22.8	5.8	0.10
Years of illicit drug use	13.9	5.0	13.5	5.1	14.6	5.0	14.2	5.0	0.07
Years of IVDA	12.6	4.3	11.3	4.3	12.8	4.3	12.4	4.4	0.12
No. of episodes of drug rehabilitation	0.7	2.4	0.6	1.8	0.9	2.8	0.8	2.5	<0.01
Duration of MMT (months)	-	-	5.0	6.9	11.4	6.9	10.7	7.0	<0.01

IVDA = intravenous drug abuse, MMT = methadone maintenance therapy

### Health insurance status

The use of health insurance and access to health insurance among participants is shown in **Table 4**. Only 46.0% of participants had health insurance, of which the most common types were voluntary insurance (34.3%) and compulsory insurance (34.5%) schemes. Regarding barriers to accessing health insurance, 36.4% of participants reported that they had insufficient information about health insurance policies; 21.0% of participants had difficulties accessing health insurance, and an additional 19.9% of patients had difficulties using health insurance. In addition, approximately 18.7% of participants had difficulty paying for health insurance. There were significant differences in health insurance among the patients from the three different levels of HIV/AIDS clinics. HIV-positive patients receiving care from provincial HIV/AIDS clinics were less likely to have health insurance (39.9% of patients from provincial clinics, vs 57.1% of patients from central clinics, p < 0.01). They were also less likely to have voluntary health insurance (23.8% of patients from provincial clinics, vs 49.1% of patients from central clinics, p<0.01). They had the highest percentage of participants on health insurance designed for the poor (23.4% of patients from provincial clinics, vs 10.01% of patients from central clinics, p<0.01), were more likely to receive inadequate information about health insurance (59.7% of patients from provincial clinics, vs 25.7% of patients from central clinics, p<0.01), were more likely to have difficulty accessing health insurance (29.1% patients from provincial clinics, vs 13.8% of patients from central clinics, p<0.01) and were more likely to have difficulty paying for health insurance (20.7% patients from provincial clinics, vs 16.5% of patients from central clinics, p < 0.01).

**Table 4:** Health insurance among participants.

	HIV/AIDS services								P-value
	Central		Provincial		District		Total		
	n	%	n	%	n	%	n	%	
**Having health insurance**									
Yes	158	57.0	109	39.9	254	43.6	521	46.0	<0.01
No	119	43.0	164	60.1	329	56.4	612	54.0	
**Number of family members with health insurance**									
0	39	14.2	57	20.9	89	15.6	185	16.5	0.01
1	43	15.6	56	20.5	150	26.3	249	22.3	
2	73	26.6	71	26.0	141	24.7	285	25.5	
3	57	20.7	49	18.0	105	18.4	211	18.9	
4	42	15.3	25	9.2	50	8.8	117	10.5	
> = 5	21	7.6	15	5.5	36	6.3	72	6.4	
**Types of health insurance**									
Voluntary	136	49.1	65	23.8	187	32.1	388	34.3	<0.01
Compulsory	93	33.6	111	40.7	187	32.1	391	34.5	0.05
Health insurance for the poor	28	10.1	64	23.4	96	16.5	188	16.6	<0.01
Health insurance for children under 6	39	14.1	46	16.9	66	11.3	151	13.3	0.08
**Inadequate Information about health insurance**									
Yes	69	25.7	160	59.7	171	30.4	400	36.4	<0.01
No	200	74.4	108	40.3	392	69.6	700	63.6	
**Difficulty accessing health insurance**									
Yes	37	13.8	78	29.1	115	20.7	230	21.0	<0.01
No	232	86.3	190	70.9	442	79.4	864	79.0	
**Difficulty using health insurance**									
Yes	51	19.2	46	17.6	119	21.4	216	19.9	0.01
No	137	51.5	123	47.1	309	55.5	569	52.5	
Unknown	78	29.3	92	35.3	129	23.2	299	27.6	
**Difficulty paying for health insurance**									
Yes	44	16.5	54	20.7	103	18.7	201	18.7	<0.01
No	191	71.8	117	44.8	328	59.5	636	59.0	
Unknown	31	11.7	90	34.5	120	21.8	241	22.4	
**Expected frequency of paying for health insurance**									
Monthly	28	10.5	19	7.1	56	10.0	103	9.4	<0.01
Quarterly	15	5.6	32	11.9	45	8.0	92	8.4	
Every six months	23	8.7	17	6.3	42	7.5	82	7.5	
Annually	183	68.8	136	50.8	343	60.9	662	60.4	
Unknown	17	6.4	64	23.9	77	13.7	158	14.4	

HI = health insurance

### Willingness to pay for monitoring tests

**Table 5** reveals that there were 1125 and 1118 patients answering questions regarding WTP for CD4 cell count and viral load tests, respectively. At the starting prices offered, only 44.7% of participants were willing to pay for CD4 cell count testing and only 15.6% were willing to pay for viral load testing.

**Table 5:** Willingness to pay for each bid among respondents.

Bid	N	%
**CD4 cell count**		
• US$ 15 (n = 1125)	503	44.7
• US$ 30 (n = 501)	149	29.7
• US$ 7.5 (n = 619)	221	35.7
**Viral load test**		
• US$ 100 (n = 1118)	174	15.6
• US$ 200 (n = 178)	38	21.4
• US$ 50 (n = 923)	163	17.8

**Table 6** summarizes WTP for CD4 cell count and viral load tests. Overall, the majority of participants were willing to pay for CD4 cell count testing (90.9% of participants) and viral load testing (87.3% of participants). Among those, the mean amount patients were willing to pay for CD4 cell count testing was US$8.2 (95%CI = 7.6–8.8 US$) per test. The mean amount patients were willing to pay for viral load testing was US$18.6 (95%CI = 16.3–20.9 US$) per test.

**Table 6:** Willingness to pay for HIV treatment monitoring—CD4 & VIRAL LOAD.

		CD4 cell count				VIRAL LOAD			
		WTP	Amount of WTP			WTP	Amount of WTP		
			(Unit: USD)				(Unit: USD)		
	N	n (%)	Mean	95%CI		n (%)	Mean	95% CI	
**All**	1133	1030 (90.9)	8.2	7.6	8.8	989 (87.3)	18.6	16.3	20.9
**Location**									
Rural	258	223 (86.4)	7.3	6.2	8.5	217 (84.1)	17.0	12.8	21.2
Urban	875	807 (92.2)	8.4	7.7	9.1	772 (88.2)	19.0	16.3	21.7
**Gender**									
Male	665	600 (90.2)	8.7	7.8	9.5	579 (87.1)	20.7	17.4	23.9
Female	468	430 (91.9)	7.5	6.5	8.4	410 (87.6)	15.6	12.5	18.6
**Current drug injection use**									
No	731	666 (91.1)	8.2	7.4	9.0	634 (86.7)	19.0	16.1	22.0
Yes	402	364 (90.5)	8.1	7.1	9.1	355 (88.3)	17.9	14.1	21.6
**Inpatient care in the last 12 months**									
No	925	839 (90.7)	7.9	7.2	8.6	807 (87.2)	16.8	14.5	19.1
Yes	208	191 (91.8)	9.3	7.8	10.9	182 (87.5)	26.2	19.2	33.2
**Outpatient care in the last 12 months**									
No	795	729 (91.7)	8.2	7.4	8.9	700 (88.1)	18.7	15.9	21.4
Yes	338	301 (89.1)	8.2	7.0	9.3	289 (85.5)	18.5	14.2	22.8
**Catastrophic healthcare expenditure in the last 12 months**									
No	868	794 (91.5)	8.2	7.5	8.9	759 (87.4)	18.5	15.9	21.1
Yes	265	236 (89.1)	8.0	6.7	9.3	230 (86.8)	18.9	13.9	23.8

(exchange rate US$ 1 = 20,000 VND)

### Factors associated with willingness to pay

The results of our reduced interval regression models are presented in **Table 7**. These regression models indicate that patients who had difficulty paying for health insurance were less willing to pay for CD4 cell counts than the other participants (p < 0.05). In addition, WTP of patients with elementary school education was significantly lower than WTP among patients with higher education levels. Respondents suffering from anxiety/depression were willing less willing to pay for testing services than those patients without anxiety/depression. Meanwhile, HIV+-positive patients who were more affluent and those who had used inpatient services in the last 12 months were willing to pay more for CD4 cell count testing (US$ 4.89 and US$ 3.39 more, respectively).

**Table 7:** Interval regressions to identify factors associated with WTP for CD4 and viral load testing.

Characteristics	Amount of WTP			
	CD4 cell count		Viral Load	
	Coef	95%CI	Coef	95%CI
**Age**	-0.16	(-0.33; 0.02)	-0.90*	(-1.63; -0.18)
**Gender** (Female vs male)	-4.69	(-10.43; 1.96)		
**Education** (vs Illiterate)				
• Elementary school	-2.85*	(-5.25; -0.45)		
• High school			5.85	(-1.91; 13.62)
• University	4.64	(-1.09; 10.36)	38.55*	(15.98; 61.13)
**Monthly income per capita (vs Poorest)**				
• Rich	4.80*	(2.45; 7.12)	7.72	(-1.28; 16.72)
**Anxiety/Depression** (Yes vs No)	-2.78*	(-5.02; -0.54)	-8.04	(-16.61; 0.54)
**Pain/Discomfort** (Yes vs No)	-2.23	(-4.51; 0.05)	-6.18	(-14.92; 2.56)
**Inpatient care in last 12 months** (Yes vs No)	3.39*	(1.03; 5.76)	9.72*	(0.58; 18.87)
**Number of episodes of drug rehabilitation**	0.26	(-0.01; 0.52)		
**Years of IVDA**	-0.16	(-4.51; 0.02)	-0.95*	(-1.81; -0.09)
**Years of illicit drug use**			0.82	(-0.19; 1.82)
**Difficulty paying for health insurance** (Yes vs No)	-3.92*	(-6.30; -1.53)	-6.3	(-15.52; 2.92)
**Difficulty accessing health insurance** (Yes vs No)			-5.66	(-14.24; 2.92)
Constant	15.95*	(9.19; 22.56)	50.34*	(24.68; 76.01)

HI = health insurance, IVDA = intravenous drug abuse, 95% confidence intervals are in parentheses. Unit: US$

* p<0.05

Patients’ WTP for viral load testing decreased remarkably with each age increase. These individuals who had a long history of IVDA were less willing to pay for viral load testing than those without a long history of injection drug use. In contrast, those patients with a university degree and those who had used inpatient services in the last 12 months were willing to pay more for viral load testing.

## Discussion

This study provides important information about WTP for CD4 cell count and HIV viral load testing among HIV-positive patients in Vietnam. Our findings reveal that a substantial percentage of the HIV-positive patients that participated in the study were amenable to paying for CD4 cell count and viral load tests, but the amount that they were willing to pay was only a fraction of the actual costs of these tests. These results suggest that continuing the HIV/AIDS disease monitoring program in Vietnam after withdrawal of foreign financial subsidies will be challenging. These findings should prompt the Vietnamese government to identify other financial sources that HIV-positive patients can use to pay for treatment monitoring services.

Using a co-payment structure to enable HIV-positive patients to affordably pay for their healthcare services is a central component of the financial sustainability of HIV/AIDS programs in Vietnam [34]. Previous studies have validated the feasibility and acceptability of co-payments for programs such as methadone maintenance treatment for HIV-positive patients [35] and HIV testing and counseling services [36]. In this study, we found that 90.9% and 87.3% of the study participants were willing to pay part of the cost of CD4 cell count and viral load tests, respectively. Respondents probably perceived the benefits of disease monitoring [36] but could not afford the full cost of either test.

Our study revealed that only 44% of participants were willing to pay for the actual cost of a CD4 cell count test in 2013; in addition, only 15.6% of patients were willing to pay for the actual cost of an HIV viral load test in 2013. The mean WTP values were US$8.2 per CD4 cell count and US$18.6 per viral load test, which were much lower than the actual cost of either test in 2013 [37]. Mean WTP values were also much lower than the current prices of the tests in more recent years (US$15 per CD4 cell count and US$40 per viral load test–according to Circular 37/2015/TTLT-BYT-BTC from the Vietnamese Ministry of Health and Ministry of Finance [38]). The costs proposed by patients for the CD4 cell count and viral load tests accounted for 0.42% and 1% respectively of the gross domestic production (GDP) per capita in Vietnam (US$ 1910 in 2013) [39].

Vietnam is currently experiencing an overall reduction in the amount of foreign aid that it receives. The country has yet to develop universal health insurance policies to fill the gap between patients’ WTP for certain health care services such as HIV monitoring tests, and the actual costs of these services [40]. A study by Tran et al. (2013) in Vietnam suggested that HIV-positive patients had to spend an annual US$188 for health care, and more than one-third of HIV-affected households could not afford these high costs [41]. Furthermore, HIV-positive patients with a long duration of co-morbid opioid abuse were less willing to pay for HIV treatment because of the concurrent high medical costs they were incurring due to their opioid dependence [24,35]. Our findings concur with previous studies that highlighted the fact that individuals with higher incomes and education levels tended to have higher WTP for health services [35,36,42,43].

Notably, participants in our study that had trouble paying for health insurance were less willing to pay for the CD4 cell count and HIV viral load tests. In this study, only 54.0% of respondents had health insurance. In addition, people receiving treatment from provincial HIV/AIDS clinics faced more difficulty accessing, using, and paying for health insurance than patients who received treatment at district or central HIV/AIDS clinics. Therefore, increasing health insurance coverage among HIV-positive patients by subsidizing insurance costs for those who are unable to pay, especially those receiving treatment at provincial HIV/AIDS clinics, is of critical importance [44,45]. Provision of health insurance, together with a co-payment structure, is necessary to ensure the overall financial sustainability of HIV/AIDS services in Vietnam [44]. Increasing the number of HIV-positive patients of health insurance would grant them coverage for both common healthcare services and HIV-related services (including clinic and laboratory visits) [45].

Our regression analysis suggests that anxiety/depression was inversely associated with WTP for monitoring HIV/AIDS treatment. This result is congruent with previous literature which has found that people with poor health conditions report lower WTP for healthcare services [35,46,47]. HIV-positive patients suffering from anxiety/depression were prone to developing hopelessness and pessimism, which led to an overall lower WTP for disease monitoring. Noticeably, in our study, the proportion of HIV-positive patients in provincial clinics who had co-morbid health problems was significantly higher than the patients who came from district or central HIV/AIDS clinics, which is consistent with previous studies [48]. The reason for this phenomenon is unclear. A recent study by Bach et al suggests a marginal association between patients in provincial clinics and non-adherence to HIV treatment [49]. Further studies are needed to better understand the unique health status of HIV-positive patients that go to provincial clinics in Vietnam for their health care.

In contrast, patients using inpatient healthcare services in the last 12 months reported higher WTP for HIV disease monitoring. There are two plausible reasons for this observation. First, patients who were recently hospitalized could develop a better awareness of the importance of disease monitoring during their hospitalization, and therefore agree to pay higher costs for these services [47,50]. Second, patients might find that regular monitoring could prevent future hospitalizations and reduce overall healthcare costs.

This study has several implications. First, our results suggest the possibility of introducing co-payment for CD4 cell count and HIV viral load tests among HIV-positive patients on ART in Vietnam, once foreign financial subsidies are withdrawn. Increasing health insurance coverage by subsidizing socioeconomically disadvantaged patients, especially those seeking treatment at provincial HIV/AIDS clinics, should be combined with co-payment schemes, to optimize the financial sustainability of these policies. Second, providing prompt health care for HIV-positive patients with physical and mental co-morbidities problems, and educating them about the merits of HIV/AIDS treatment monitoring, may raise patients' overall WTP.

This study has several strengths. First, we included a large sample size of HIV+-positive patients, which improved the validity and reliability of our measurements. Additionally, to make the sample more representative, we selected a range of HIV/AIDS health clinics, located in both urban and rural areas and from different healthcare levels (central, provincial and district level HIV/AIDS services). Nevertheless, several limitations were present in this study. First, our cross-sectional design could not establish cause and effect between WTP for HIV disease monitoring and other factors such as access to health insurance. Second, the contingent valuation approach may not completely reflect what patients would pay for tests when they are actually required to fund the tests themselves. By using the DBDC method, respondents may be uncomfortable if they believed they had struck a deal with their response to the first question but were then asked a follow-up question with a different dollar amount. They might also have felt guilty about saying no to the first amount, and might have felt compelled to say yes to the second (smaller) amount. Third, the data analyzed in this study was based on self-reported information, which is subject to recall bias. There is also possible bias introduced if patients believe particular responses are more socially desirable and are what the interviewer might have wanted to hear. For example, participants may not have been willing to pay for CD4 cell count and viral load testing, but may have responded that they were willing to pay for the test if they believed the interviewer wanted them to answer in that way. Such biases could lead to under- or over-estimation of patients’ WTP for these services. Fourth, the dollar amount set to evaluate patients’ WTP in this study was hypothetical, highlighting the need for further studies to better elucidate WTP in the actual clinical setting before implementation [47]. Finally, because we used a convenience sampling approach, our findings might not be generalizeble to other settings and patient populations.

## Conclusion

In conclusion, our findings suggest that HIV-positive patients in Vietnam have high rates of co-morbid health problems and overall low rates of health insurance coverage, especially if they are receiving treatment at provincial clinics. HIV-positive patients were only afford small co-payments for monitoring of their disease progression. The Vietnamese government needs to ensure feasibility of sustainable financing policies to help HIV-positive patients afford routine monitoring services and thereby improve their health outcomes. Removing barriers to accessing health insurance may narrow the gap between patients’ WTP and the actual costs of monitoring tests. HIV-positive patients receiving treatment from provincial HIV/AIDS centers faced the greatest difficulty in acquiring health insurance.
